# Supplementary material for: The Effect of Acceptor Structure on Emission Color Tuning in Organic Semiconductors with D–π–A–π–D Structures
Source: Nanomaterials (Basel). 2019 Aug 17;9(8):1179. doi: 10.3390/nano9081179 (PMC6724117; doi:10.3390/nano9081179)
Supplement: Supplementary file 1 [file nanomaterials-09-01179-s001.pdf]

Electronic Supplementary Information (ESI)

The effect of acceptor structure on emission colour tuning in organic semiconductors with D-π-A-π-D structures

Przemyslaw Ledwon[1,*], Gabriela Wiosna-Salyga[2,*], Marian Chapran[2] and Radoslaw Motyka[1]

[1] Silesian University of Technology, Faculty of Chemistry, Strzody 9, 44-100 Gliwice, Poland;
[2] Department of Molecular Physics, Lodz University of Technology, Zeromskiego 116, 90-924 Lodz, Poland;
* Correspondence: przemyslaw.ledwon@polsl.pl; Tel.: +48-32-237-1305 (P.L.); gabriela.wiosna-salyga@p.lodz.pl; Tel.: +48 42-631-3205 (G.W.-S.)

Table of content

Figure S1-S13: 13C-NMR and 1H-NMR spectra p. 2-9

Figure S16. UV-Vis and photoluminescence spectra spectra of studied compounds, measured in different solvents p .10

Figure S17. Photoluminescence spectra of emitters in the different solvents, 1 wt% in zeonex matrix and in pristine films p. 11

Figure S18. Photoluminescence decay curves of studied compounds in the different solutions (10−5 M in toluene, chloroform, THF, DCM) at the maximum emission p. 12

Table S1. The fluorescence lifetime of the red emitters in diluted solutions obtained from photoluminescence decay curves at the maximum emission p.12

Figure S19. Normalized absorption spectra of the emitters in toluene solution and photoluminescence spectrum of PVK:PO-T2T in film p. 13

Figure S20. Normalized photoluminescence spectra of thin layers: PVK:PO-T2T doped with 1, 2, 5, 8, 10 wt% of studied molecules. p. 14

NMR Spectra

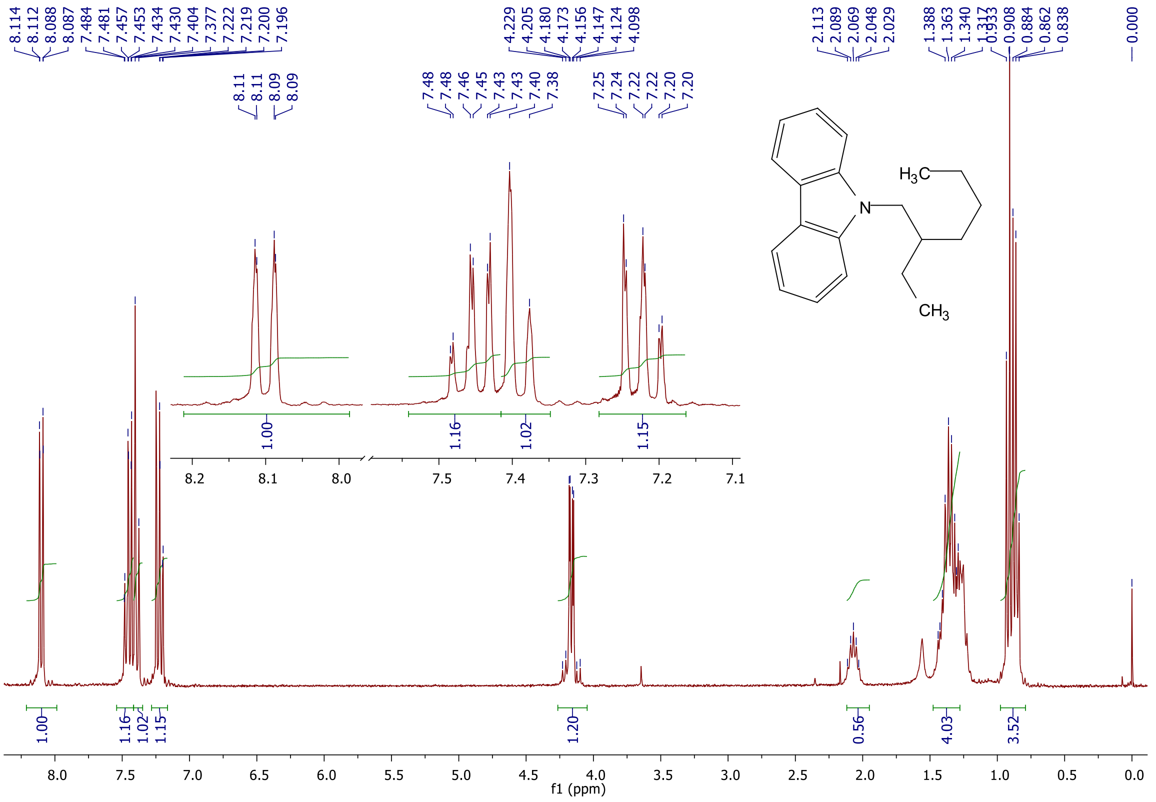

Figure S1. ^{1}H-NMR of compound **2** in $CDCl_3$

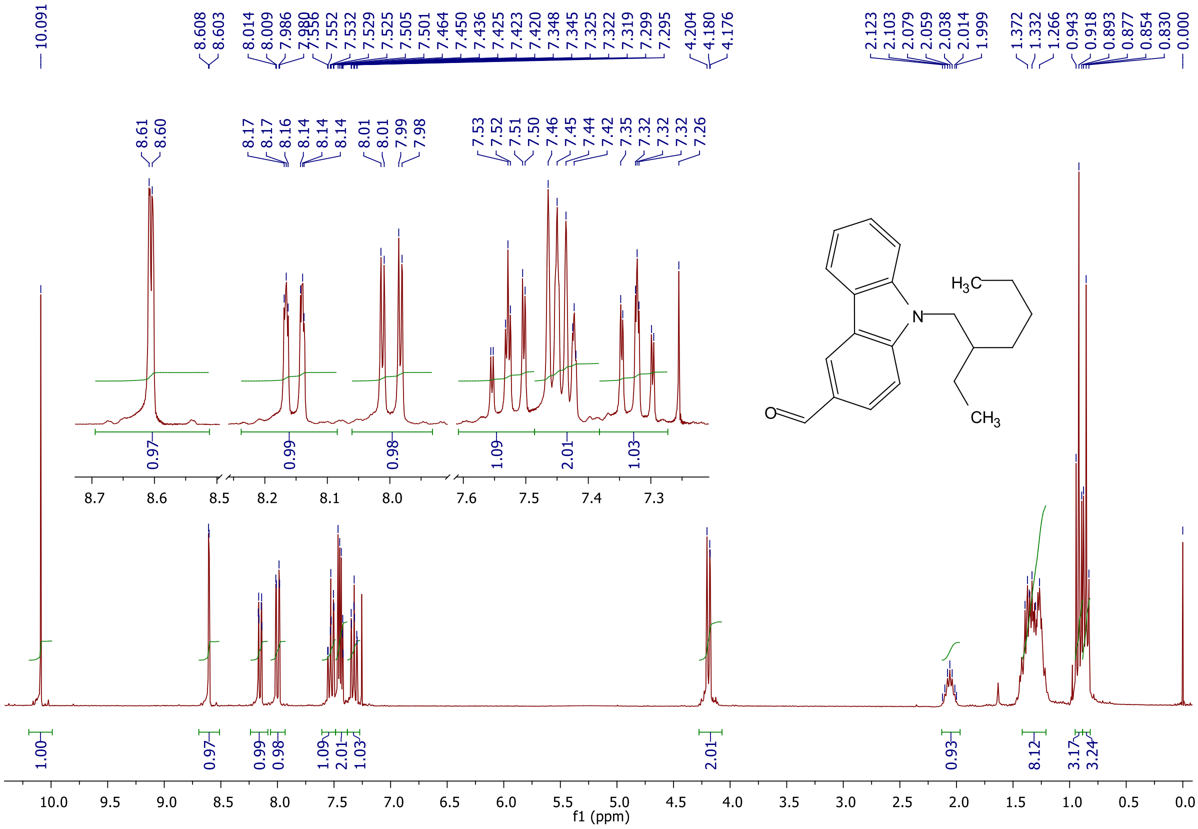

Figure S2. ^{1}H-NMR of compound **3** in $CDCl_3$

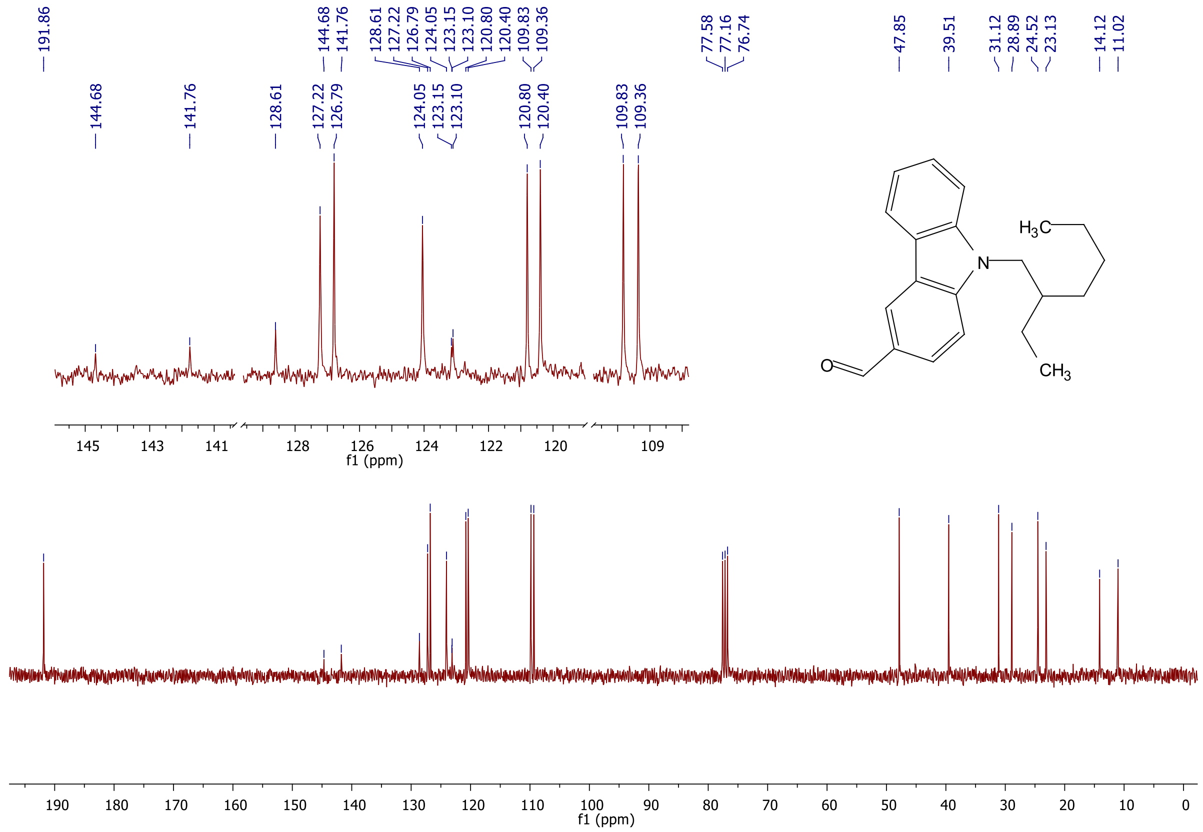

Figure S3. ^{13}C-NMR of compound **3** in $CDCl_3$

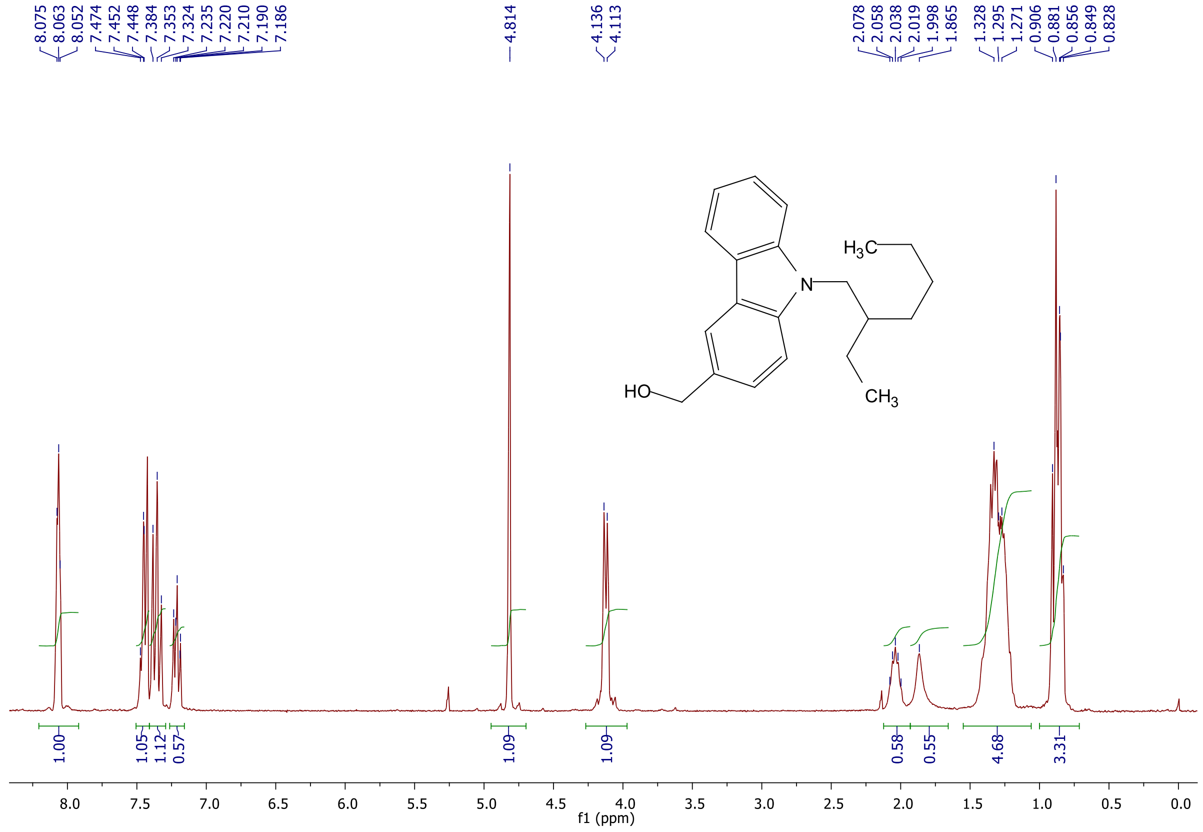

Figure S4. ^{1}H-NMR of compound **4** in $CDCl_3$

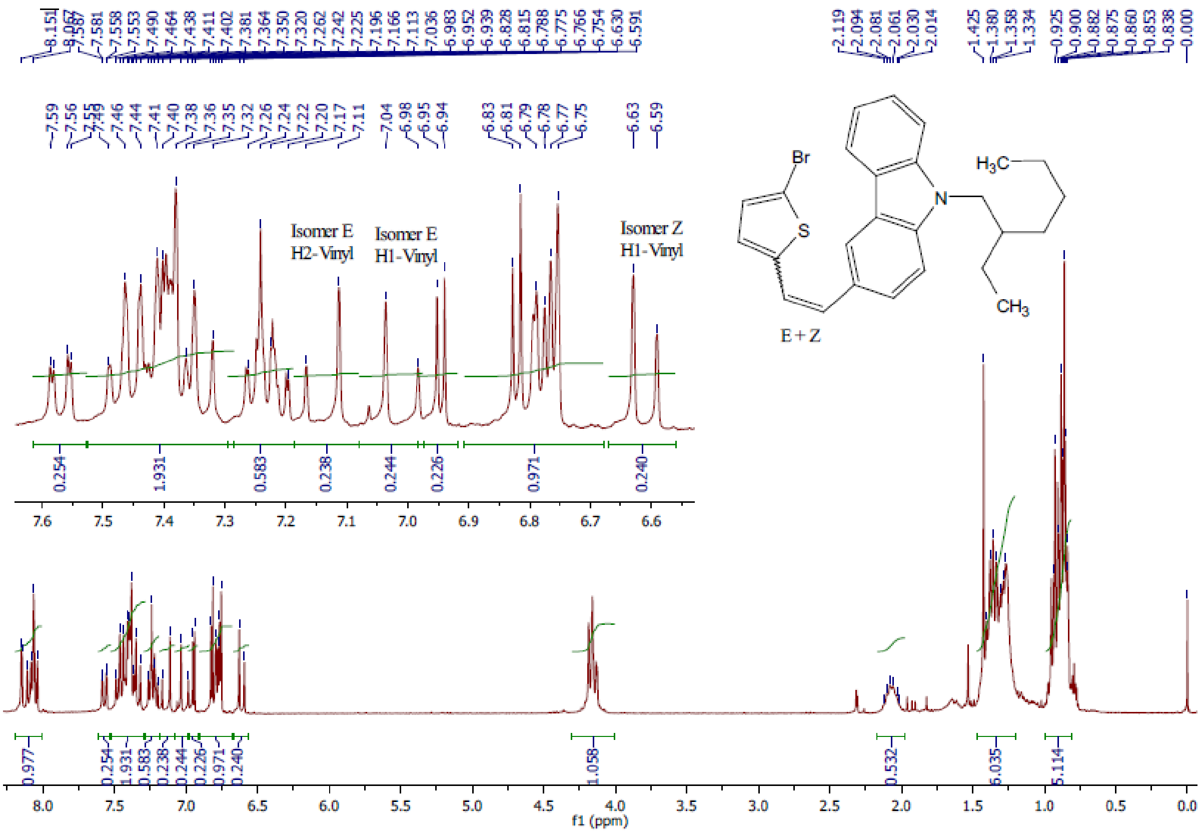

Isomer E
H2- Vinyl
Isomer E
H1- Vinyl
Isomer Z
H1- Vinyl
Br
S
N
H_3C
CH_3
E + Z
f1 (ppm)

Figure S5. ^{1}H-NMR of mixture of compounds **6** in $CDCl_3$

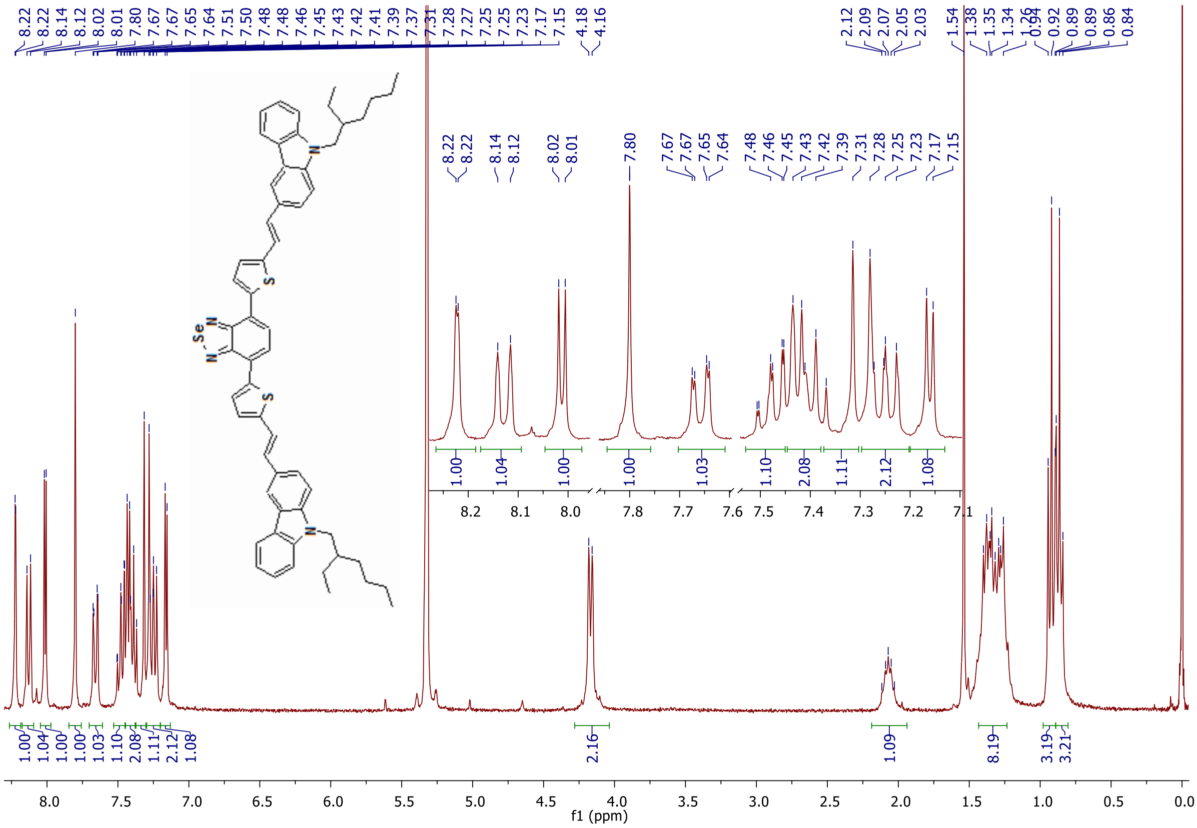

Figure S6. ^{1}H-NMR of compound **C4** in CD_2Cl_2

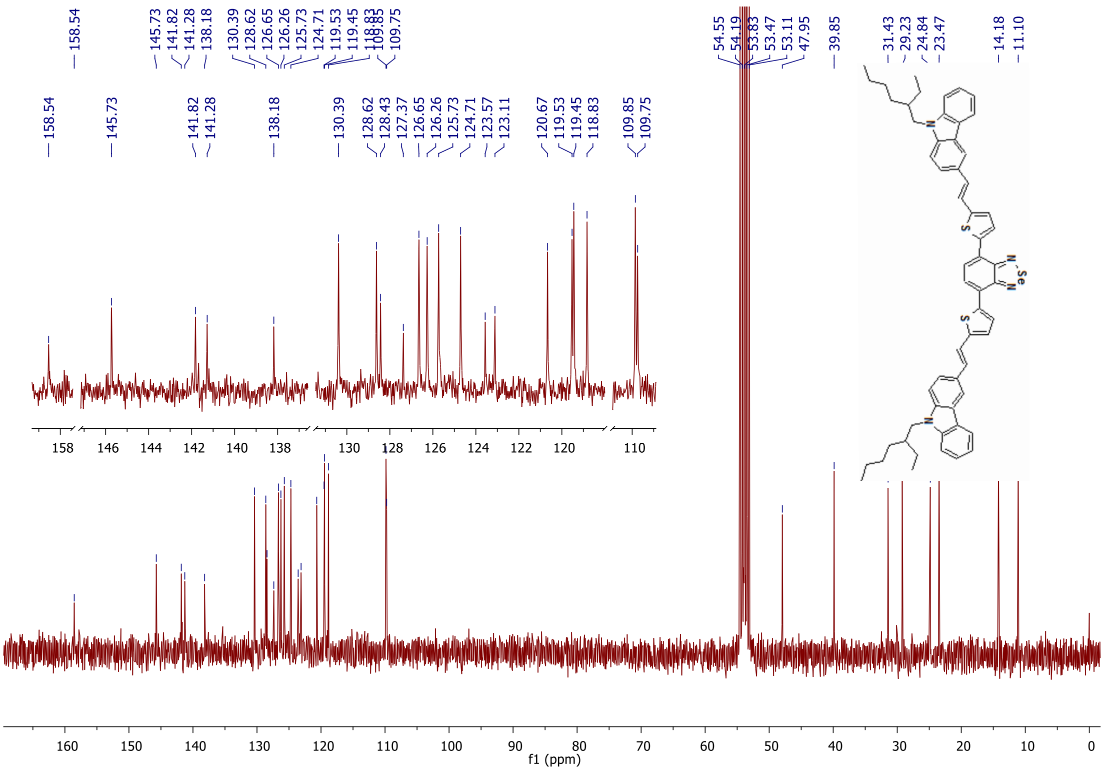

Figure 7. ^{13}C-NMR of compound **C4** in CD_2Cl_2

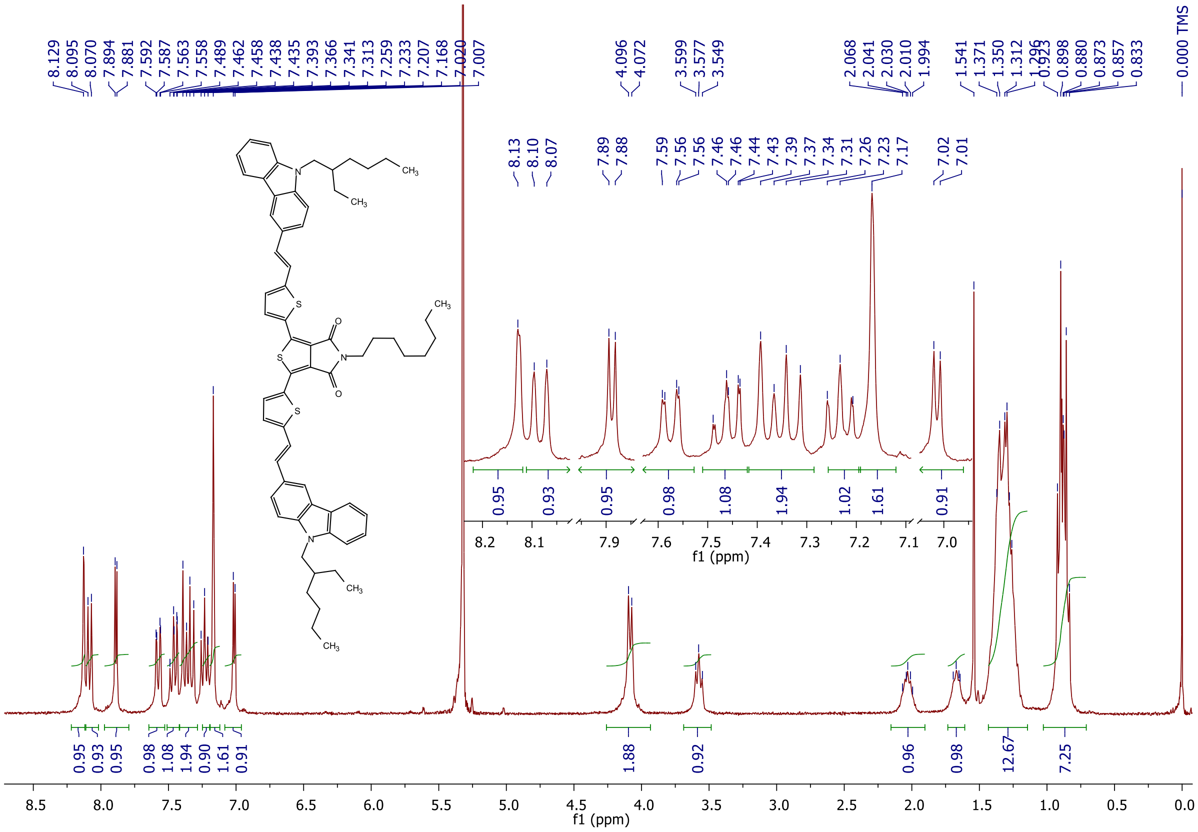

Figure S8. ^{1}H-NMR of compound **C1** in CD_2Cl_2

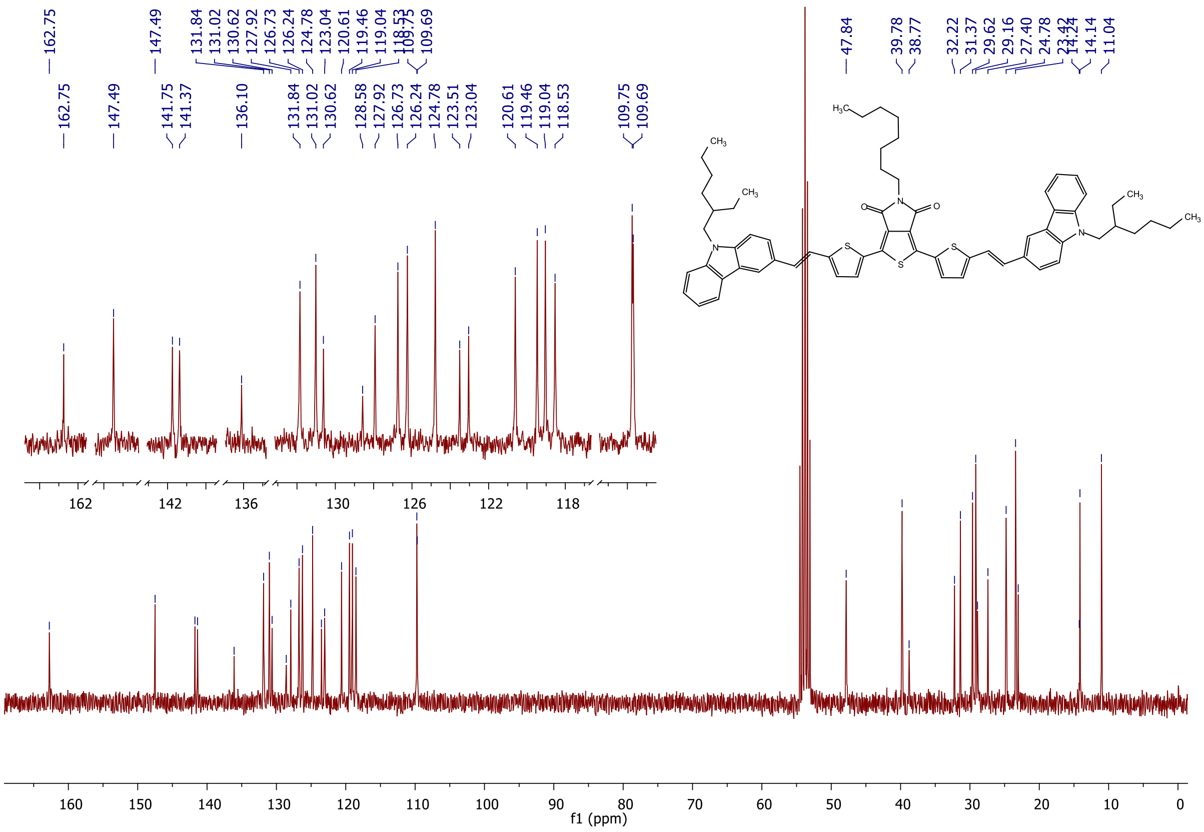

Figure S9. ^{13}C-NMR of compound **C1** in CD_2Cl_2

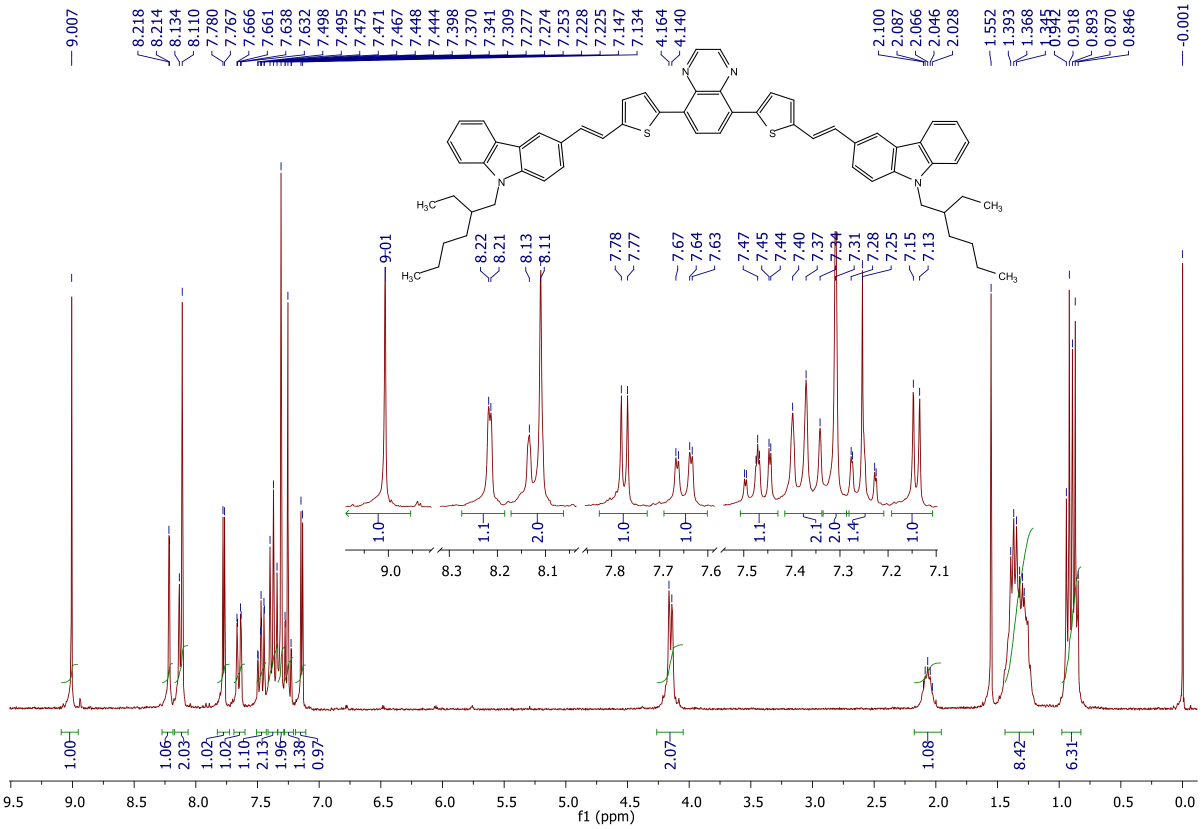

Figure S10. ^{1}H-NMR of compound **C2** in $CDCl_3$

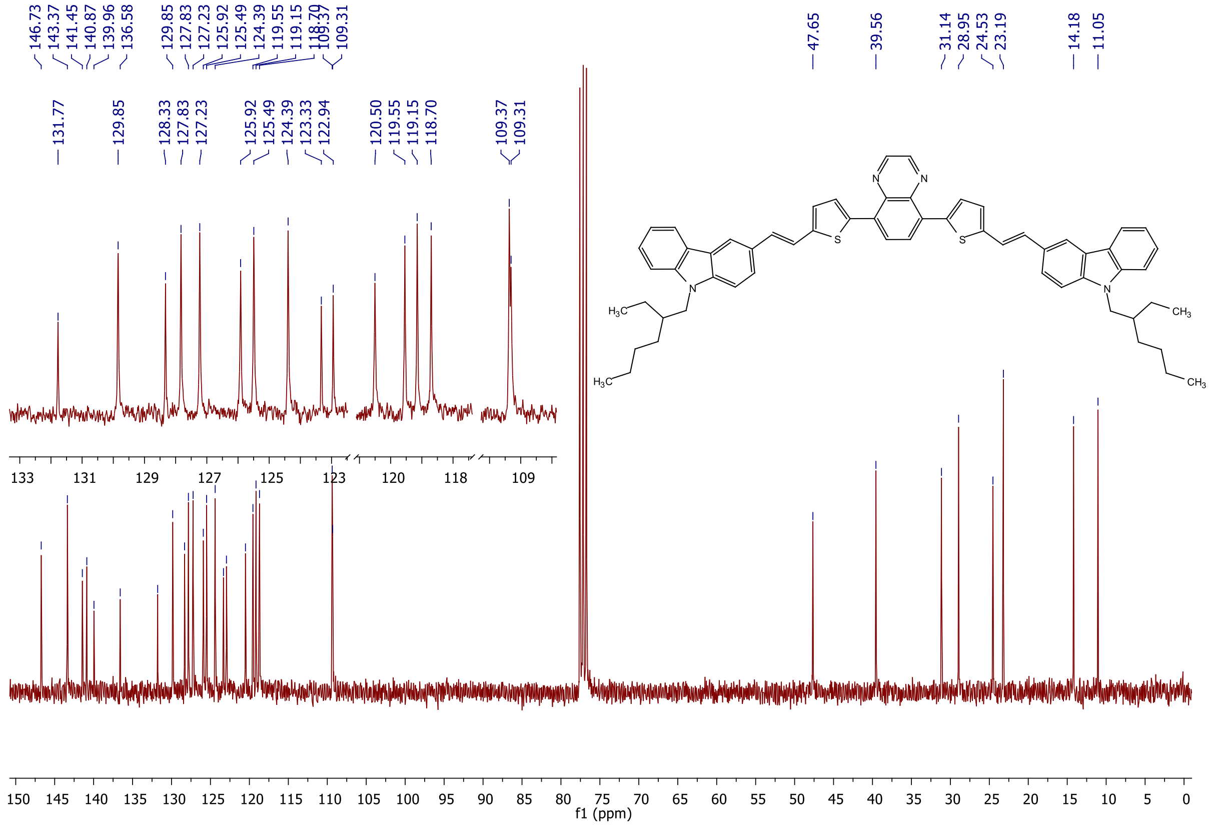

Figure S11. ^{13}C-NMR of compound **C2** in $CDCl_3$

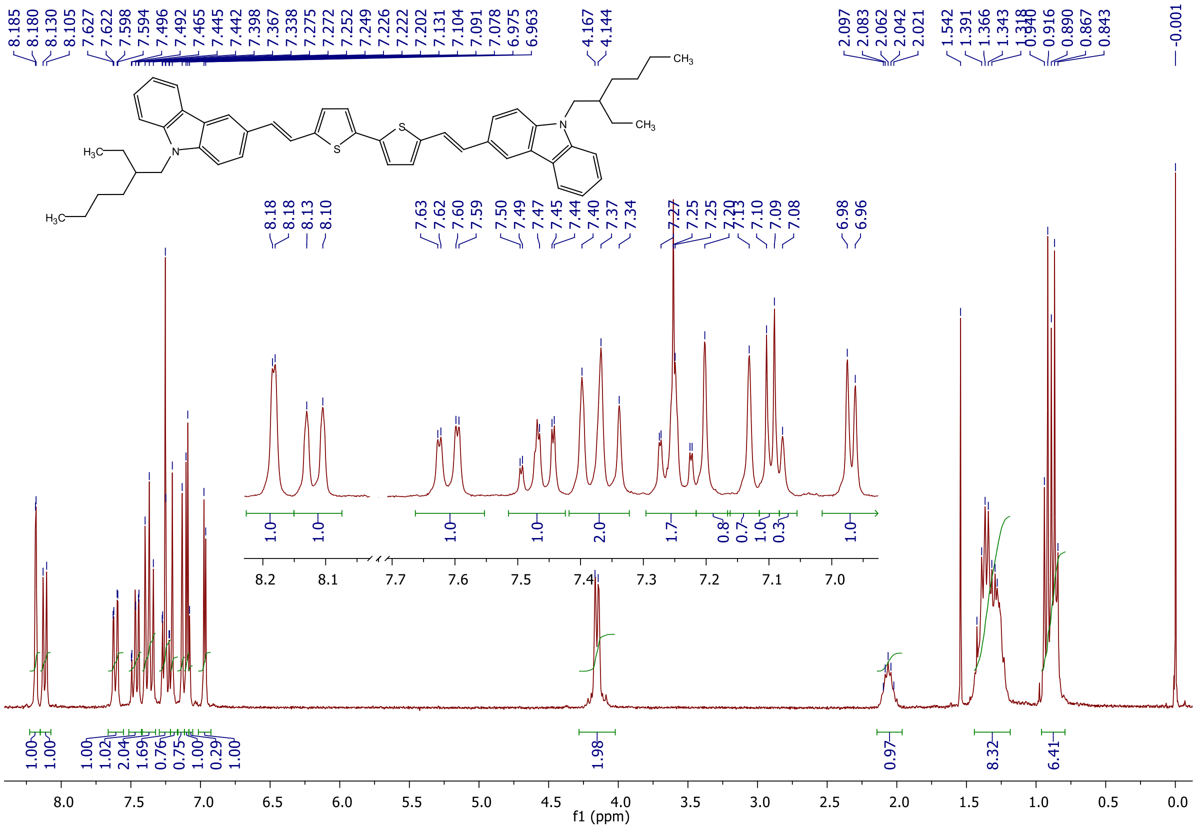

Figure S12. ^{1}H-NMR of compound **C5** in $CDCl_3$

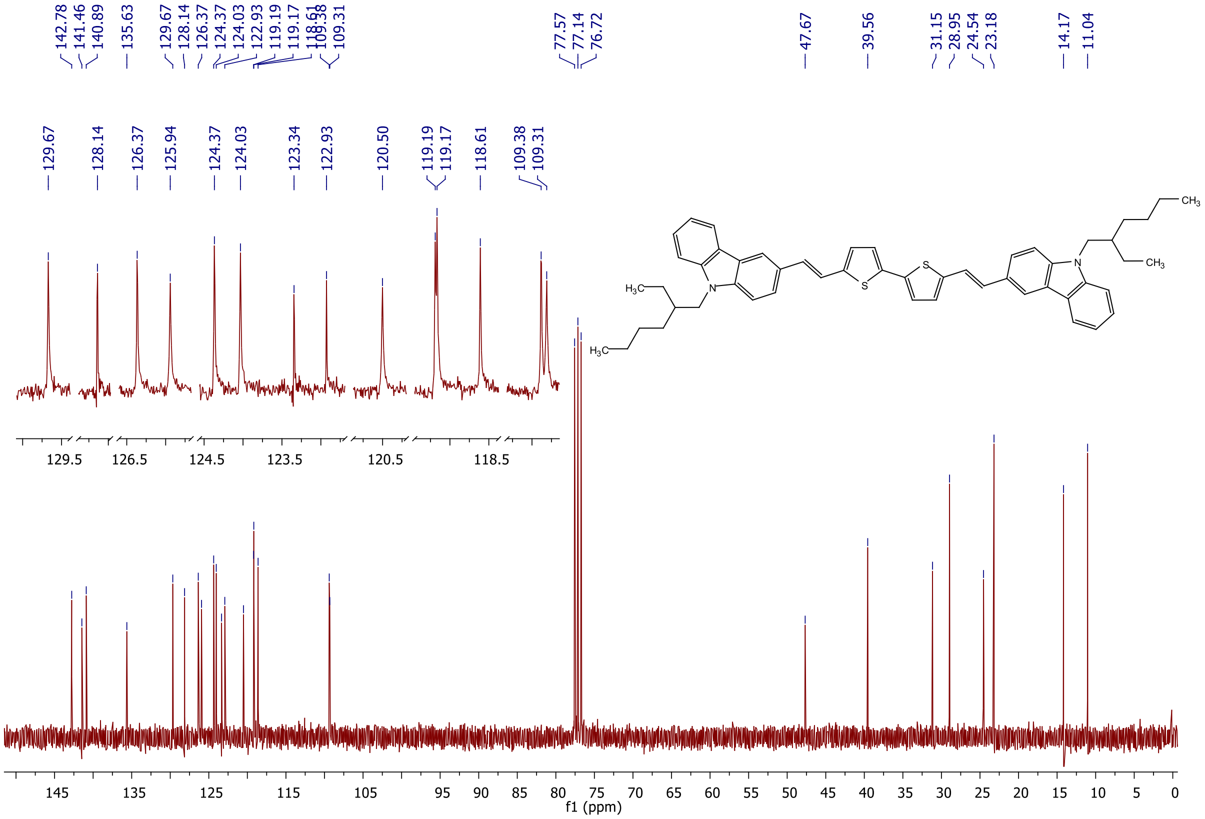

Figure S13. ^{13}C-NMR of compound **C5** in $CDCl_3$

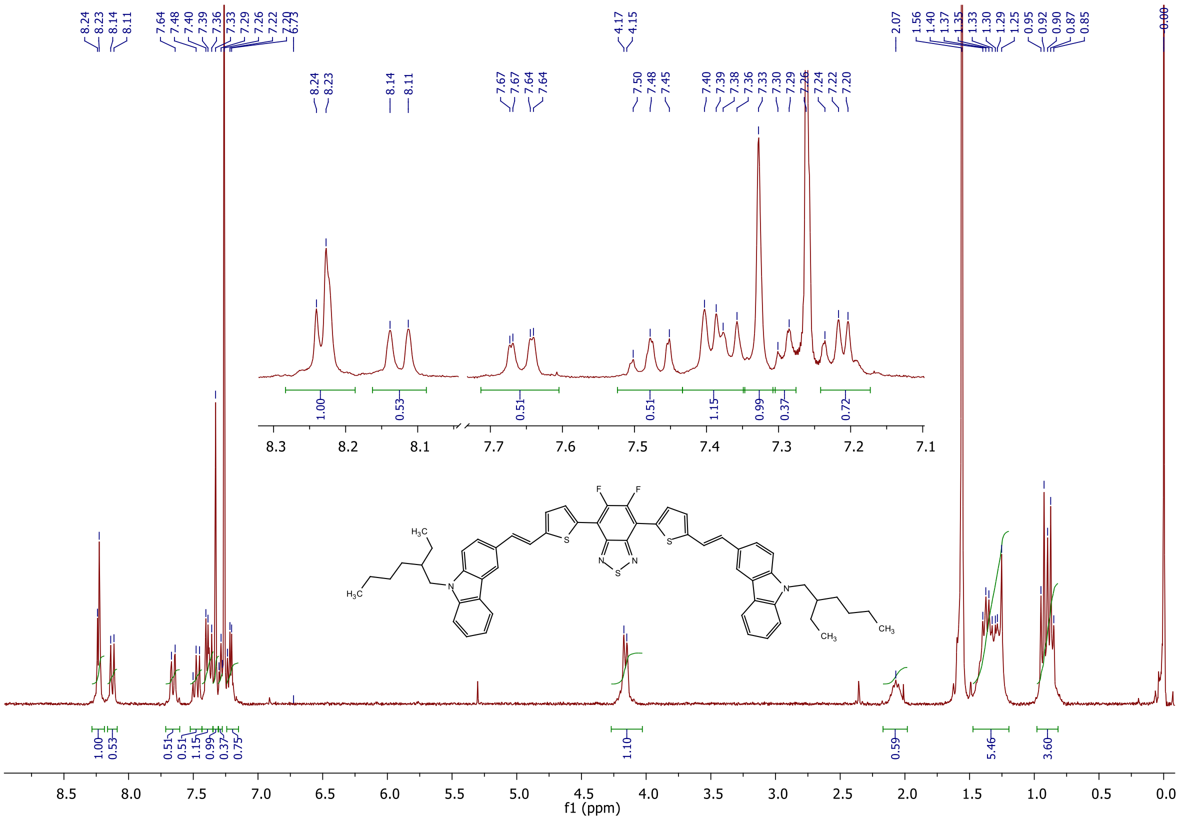

Figure S14. ^{1}H-NMR of compound **C3** in $CDCl_3$

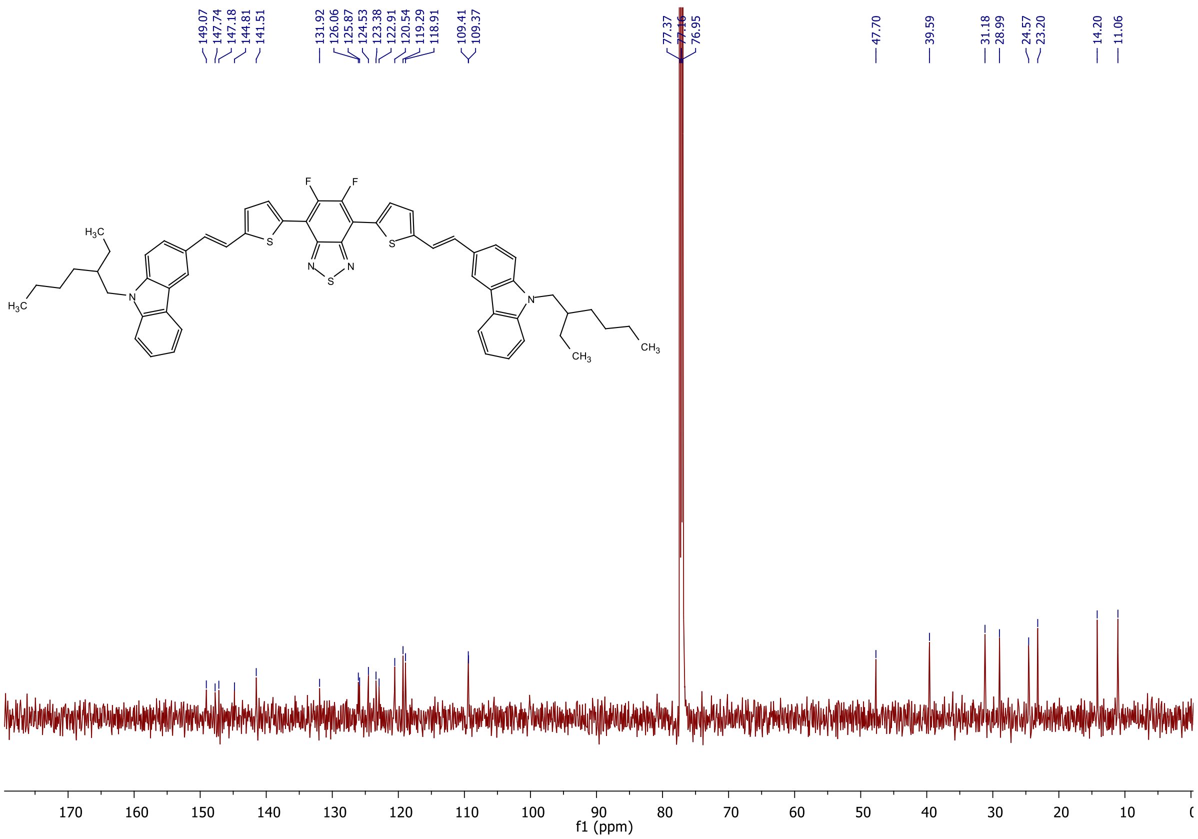

Figure S15. ^{13}C-NMR of compound **C3** in $CDCl_3$

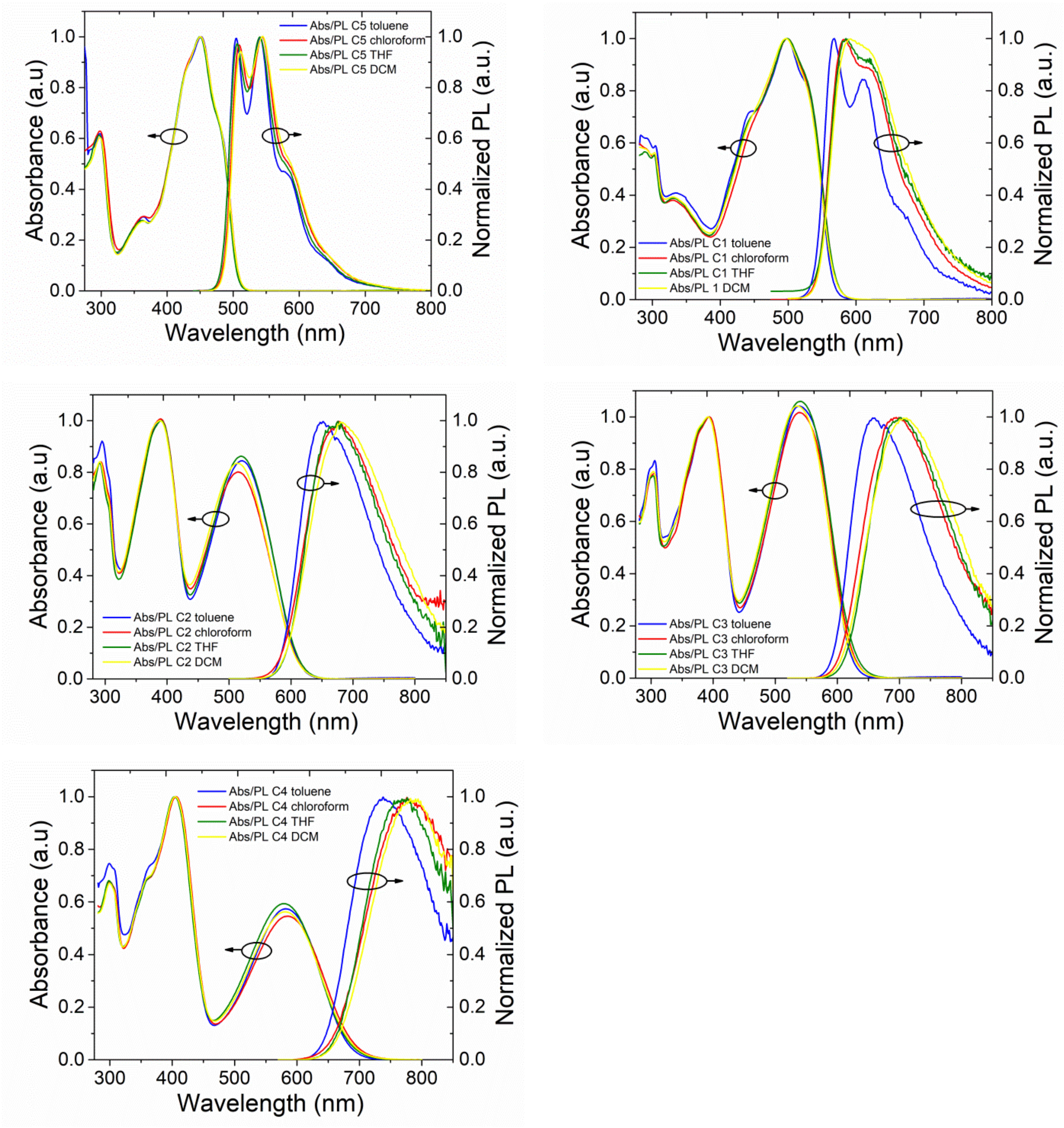

Figure S16. UV-Vis and photoluminescence spectra spectra of studied compounds, measured in different solvents.

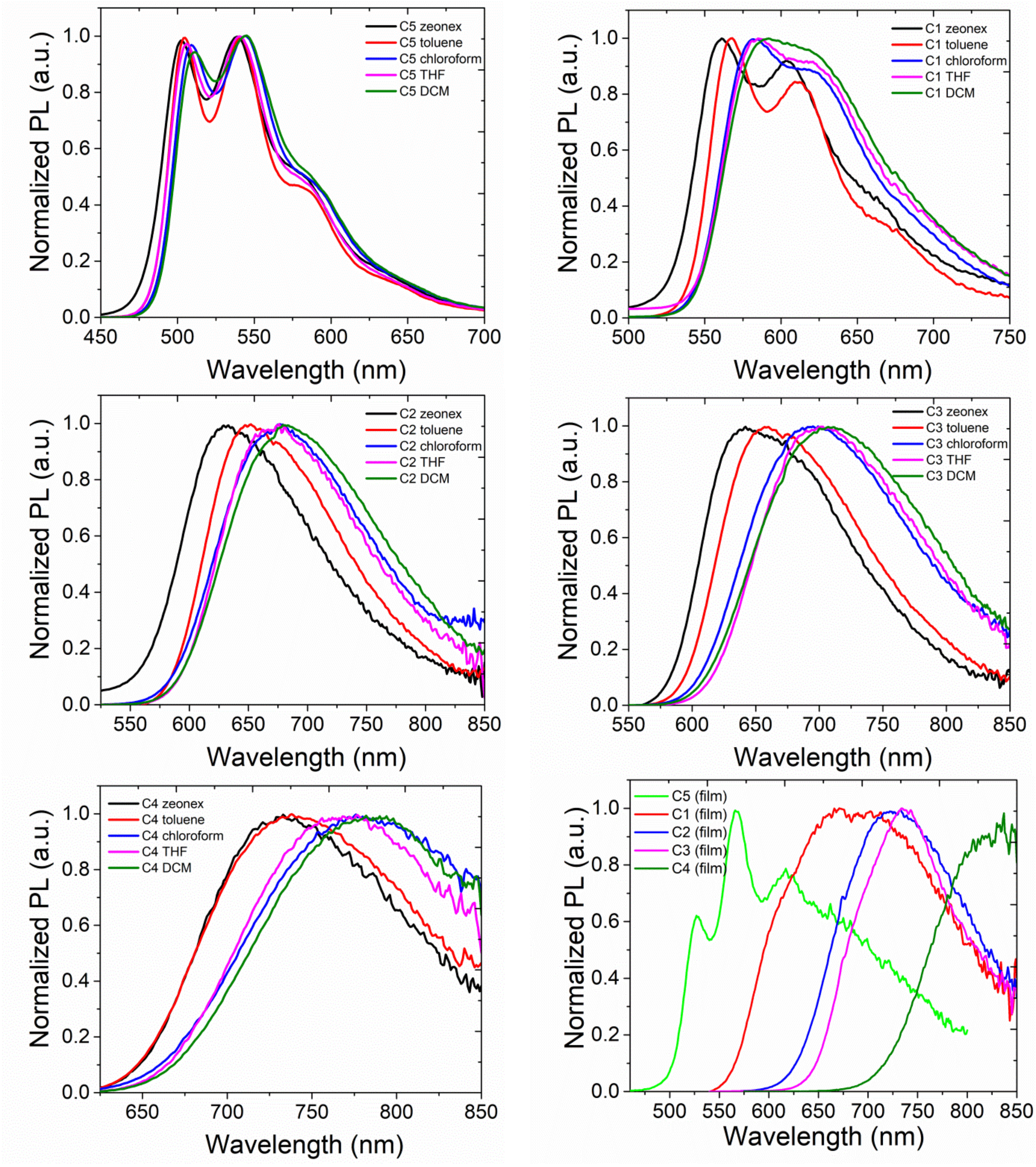

Figure S17. Photoluminescence spectra of emitters in the different solvents, 1 wt% in zeonex matrix and in pristine films.

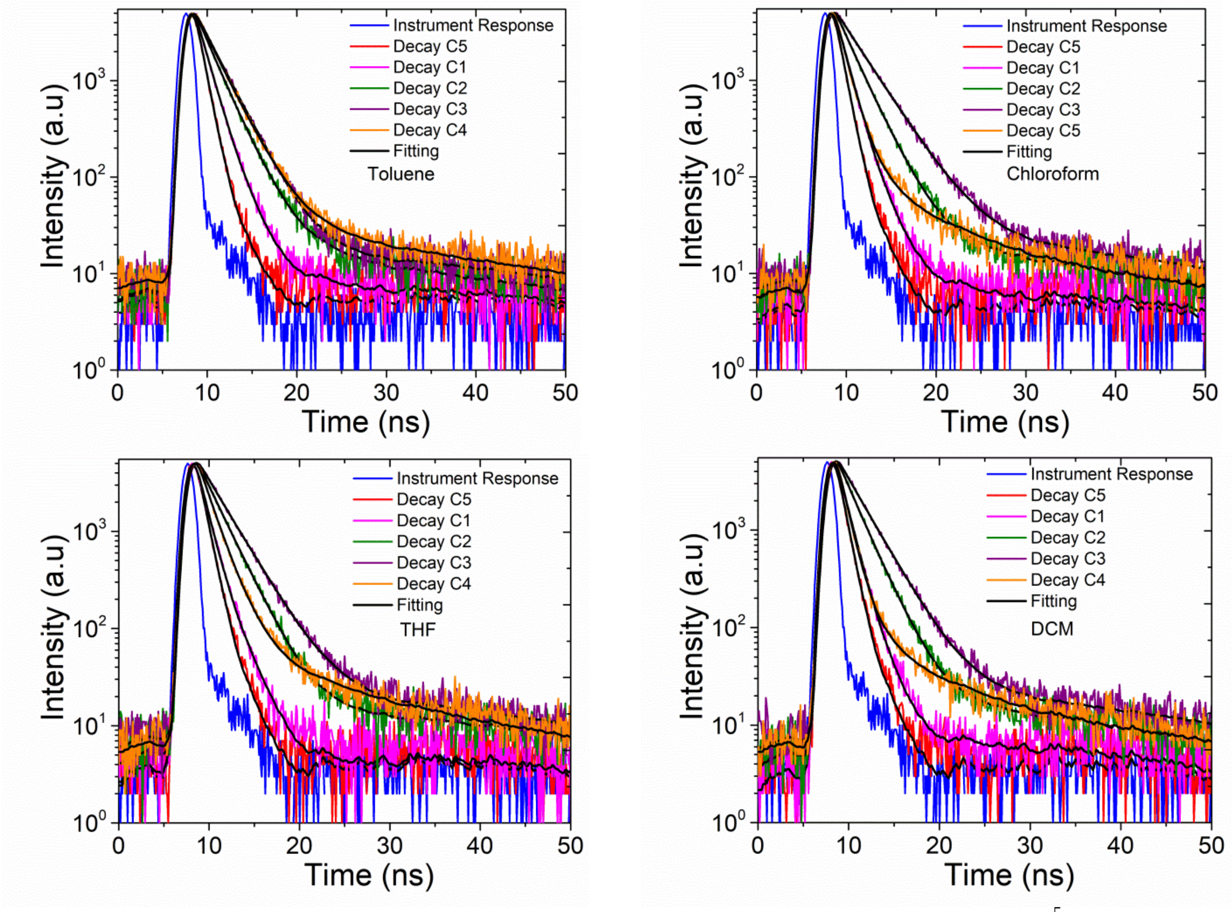

Figure S18. Photoluminescence decay curves of studied compounds in the different solutions (10^{-5} M in toluene, chloroform, THF, DCM) at the maximum emission.

Table S1. The fluorescence lifetime of the red emitters in diluted solutions obtained from photoluminescence decay curves at the maximum emission

	Toluene			Chloroform			THF			DCM		
	τ_1 ns (%)	τ_2 ns (%)	χ^2	τ_1 ns (%)	τ_2 ns (%)	χ^2	τ_1 ns (%)	τ_2 ns (%)	χ^2	τ_1 ns (%)	τ_2 ns (%)	χ^2
C5	0.9(100)	-	1.28	0.8(100)	-	1.42	0.9(100)	-	1.32	0.9	-	1.36
C1	1.3 (97)	6.1 (3)	1.28	1.2 (98)	5.7 (2)	1.29	1.1 (91)	2.3 (9)	1.52	1.1 (98)	14.9 (2)	1.25
C2	1.9 (95)	8.3 (5)	1.29	2.1 (96)	9.8 (4)	1.21	2.1 (97)	18.5 (3)	1.33	1.9 (97)	13.8 (3)	1.29
C3	2.3 (97)	16.6 (3)	1.28	2.9 (96)	14.2 (4)	1.28	2.7 (97)	15.7 (3)	1.28	2.5 (97)	16.2 (3)	1.21
C4	2.2 (95)	11.2 (5)	1.14	1.1 (91)	8.2 (9)	1.48	1.5 (93)	9.7 (7)	1.27	1.0 (92)	8.6 (8)	1.27

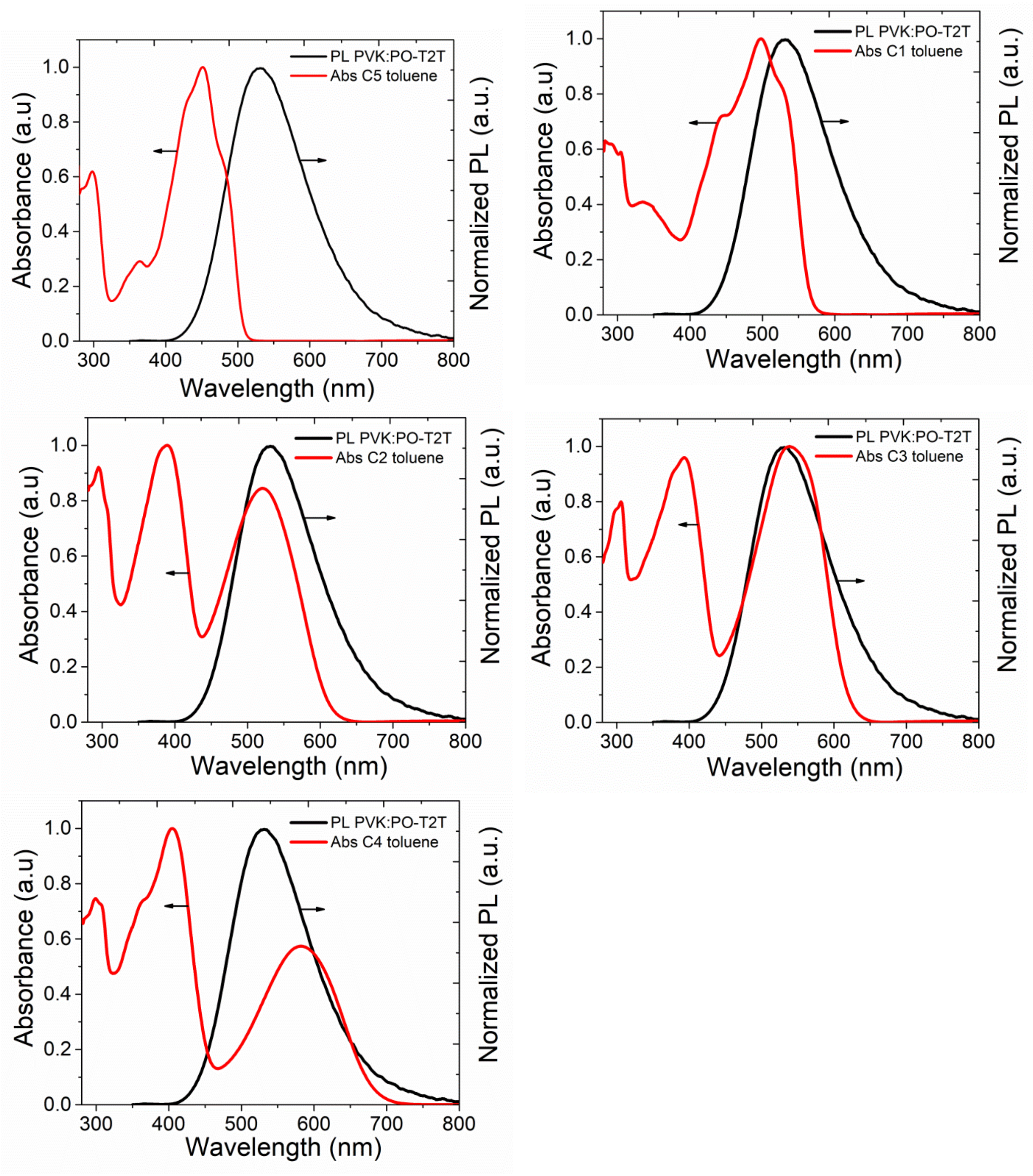

Figure S19. Normalized absorption spectra of the emitters in toluene solution and photoluminescence spectrum of PVK:PO-T2T in film.

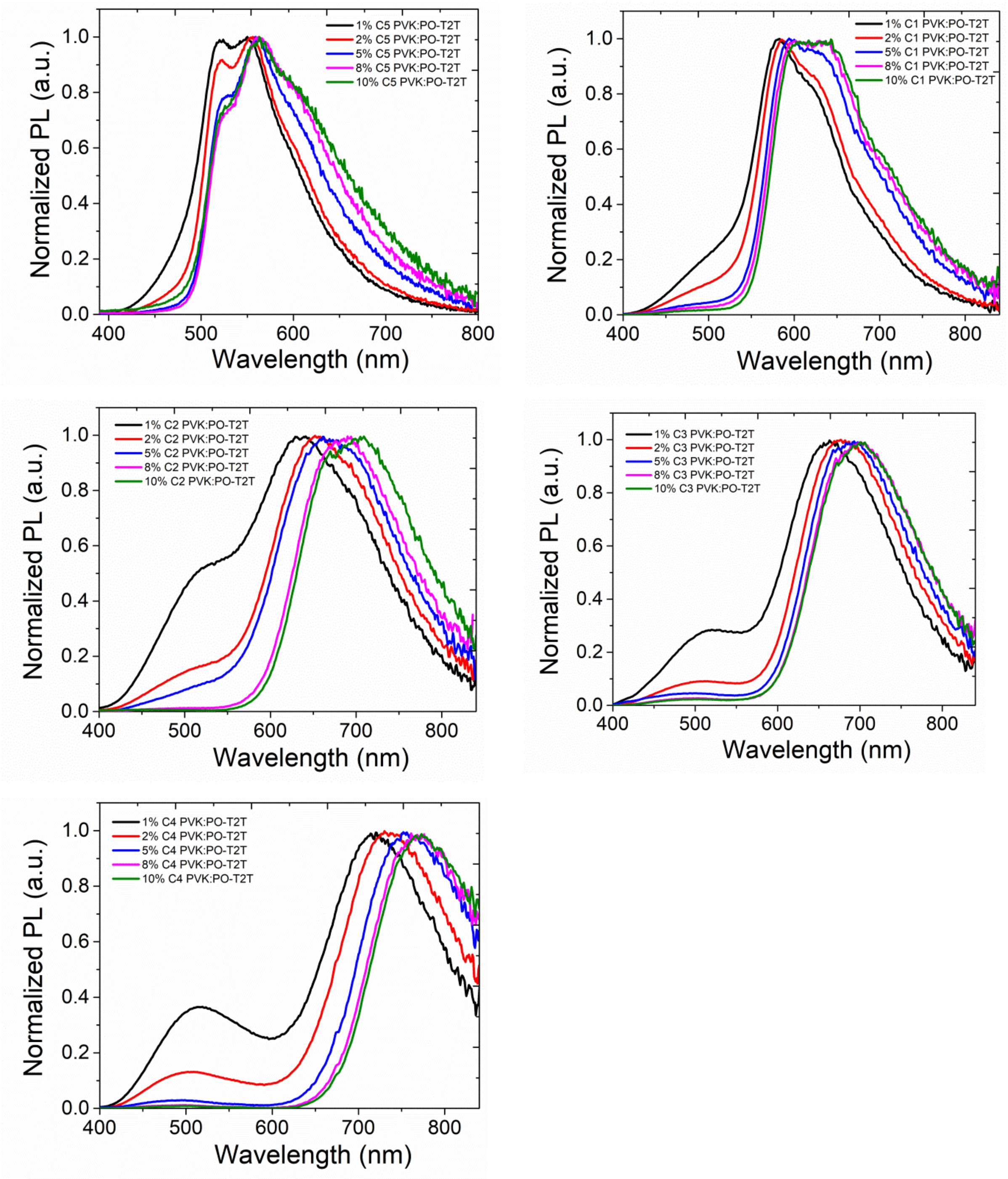

Figure S20. Normalized photoluminescence spectra of thin layers: PVK:PO-T2T doped with 1, 2, 5, 8, 10 wt% of studied molecules.
